# Sociodemographic, behavioral, obstetric, and healthcare factors associated with low weight at birth: a case-control study

**DOI:** 10.1590/1516-3180.2022.0615.R1.24042023

**Published:** 2023-07-10

**Authors:** Viviane Tazinasso Cluzeni, Guilherme Welter Wendt, Lirane Elize Defante Ferreto, Léia Carolina Lucio, Claudicéia Risso-Pascotto

**Affiliations:** I MSc. Nutritionist and Student, Postgraduate Program of Applied Health Sciences, Universidade Estadual do Oeste do Paraná (UNIOESTE), Francisco Beltrão (PR), Brazil.; II PhD. Psychologist and Adjunct Professor, Health Sciences Center, Universidade Estadual do Oeste do Paraná (UNIOESTE), Francisco Beltrão (PR), Brazil.; III PhD. Pharmacist, Associate Professor, Health Sciences Center, Universidade Estadual do Oeste do Paraná (UNIOESTE), Francisco Beltrão (PR), Brazil.; IV PhD. Biologist and Associate Professor, Health Sciences Center, Universidade Estadual do Oeste do Paraná (UNIOESTE), Francisco Beltrão (PR), Brazil.; V PhD. Biologist and Associate Professor, Health Sciences Center, Universidade Estadual do Oeste do Paraná (UNIOESTE), Francisco Beltrão (PR), Brazil.

**Keywords:** Birth weight, Pregnancy, Prenatal care, Infant, Public policy, Infant care, Maternal-child health, Low weight at birth, Health in pregnancy

## Abstract

**BACKGROUND:**

Understanding social determinants is crucial for implementing preventive strategies, especially for low birth weight (LBW)—a public health issue that severely increases the risk of morbimortality in children.

**OBJECTIVE:**

This study aimed to identify the factors associated with LBW among newborns, assisted by the Brazilian Unified Health System.

**DESIGN AND SETTING:**

It analyzed data from newborns and their mothers. The sample was selected by convenience from users of the public health system in Francisco Beltrão (Paraná, Brazil).

**METHODS:**

Cases (n = 26) were babies weighing ≤ 2,500 g and controls (n = 52) > 2,500 g. All babies were assessed and paired by sex and date of birth in a 1:2 proportion. Statistical power was computed a posteriori, revealing a power of 87% (α = 0.05).

**RESULTS:**

Strong and significant differences were found in the bivariate analysis, in which the number of current smokers or those who quit during pregnancy was higher among mothers of babies with LBW. Moreover, the gestational weeks were lower among these cases. Logistic regression models indicated that the gestational week (odds ratio [OR] = 0.17, 95% confidence interval [CI]:0.05–0.54) and fathers’ educational level (high school or above; OR = 0.22, 95% CI:0.06–0.99) were related to lower chances of low birth weight.

**CONCLUSIONS:**

Our findings confirm previous investigations on LBW's multi-causality, showing that the gestational week could reduce up to 82% chances of a baby being born with ≤ 2,500 g. Its association with paternal education underlines the importance of comprehensive policies to protect newborns.

## INTRODUCTION

This study explores the factors associated with low birth weight (LBW) in newborns assisted by the Brazilian Unified Health System (Sistema Único de Saúde [SUS]). Compared with babies with regular weights, LBW newborns are up to 20 times more likely to die, and preventive efforts include myriad factors.^1,2^ The present investigation focused on the sociodemographic, behavioral, obstetric, and healthcare variables underpinning LBW.

For over a century, healthcare professionals have considered newborn weight a parameter for infant care and mortality. The 2,500 g cutoff value for LBW was first set in 1919, when the difference between LBW and prematurity was not clear-cut.^3^ LBW increases the chances of cardiovascular diseases, diabetes, and cognitive deficits during the baby's life.^1,2^ Thus, it is understood as a public health issue, guiding the development of health actions and setting parameters for the number of neonatal intensive care.^4^

Despite its association with social vulnerability, LBW occurs in both developed and developing countries. In Brazil, the incidence is around 8.5%, which is similar or slightly inferior to data from the state of Paraná.^5^ Several factors may be at play in LBW, wherein the most cited ones are the precocious inducement of birth by cesarean section, multiparity, comorbidities, and the pregnant woman's lifestyle.^2^ Preterm births may increase the risk of LBW by up to 35 times when compared to term babies.^6^

Behavioral habits, nutritional factors, smoking, and the use of illicit drugs are risk factors for LBW and should be the focus of interventions. Maternal obesity is responsible for complications for the mother, fetus, and during perinatal periods, and it must be controlled in prenatal care.^7^ Even in women with eutrophic pregestational weight, controlling weight gain during pregnancy is essential to reduce diseases and their aggravation.^8,9^ Evidence warns the effects of the habit and exposure to tobacco smoke in the uterine environment and postnatal period, and its relationship with LBW and several adverse short- and long-term effects, including congenital anomalies, miscarriages, behavioral syndromes, and even childhood cancer.^1,10^ Illegal drug use is harmful in a handful of ways, among which, the reduction of fetal weight gain is significant.^11^

A pregnant woman's external environment directly influences her health status and gestational outcomes. Factors such as income, age, age during her first pregnancy, number of pregnancies, education, occupation, marital status, and social situation are strongly associated with quality of life during pregnancy and LBW.^12^ However, the risks and protective factors are not only putative maternal characteristics but also paternal influences,^13^ and low educational attainment could constitute a risk factor. Nevertheless, we found no studies connecting partners’ education with LBW.

One of the most effective ways to minimize the risks involved in pregnancy and LBW is to assist all women of reproductive age through family planning. As advised by the World Health Organization, quality prenatal care must include at least six physician appointments and begin as early as possible, preferably before the 12^th^ gestational week. The Basic Units of Health (in Portuguese, Unidade Básica de Saúde [UBS]) are the first spaces for sheltering pregnant women and screening for possible gestational risks associated with LBW.^14^

Understanding the social determinants (exposure outcomes) is crucial for implementing preventive strategies, especially in the case of LBW, a public health issue that severely increases the risk of morbimortality.^15^ Currently, there are few case-control studies^16,17^ that broadly evaluate the individual contributions of various exposure factors connected to LBW, such as sociodemographic, behavioral, obstetric, and healthcare characteristics.

## OBJECTIVES

The present research sought to compare risk factors associated with LBW, as well as to provide useful information for healthcare professionals and policymakers involved in maternal and infant health, by investigating a far-reaching group of factors and outcome data of newborns. The main hypothesis was that in the sociodemographic dimension, parents’ elevated incomes and higher education levels would result in lower chances of LBW,^13^ while behavioral risk factors (such as smoking and using drugs) would increase LBW chances.^1,10,11^ Based on other investigations, it was also estimated that access to healthcare – measured by the early start and high number of prenatal appointments – would constitute a protective factor.^14^

## METHODS

This community-based case-control study^18^ analyzed data from newborns and their mothers. The initial population consisted of 432 pregnant women selected by convenience among users of the public health system in Francisco Beltrão (Paraná, Brazil) between July 2018 and July 2019.

During this period, 26 babies born weighing ≤ 2,500 g were considered for this study. Controls were defined as term babies weighing > 2,500 g. Controls were selected in a 2:1 ratio and paired according to their sex and birth date. This was performed to reduce any bias regarding sex differences in terms of risks for mortality, as well as to account for environmental and other external factors that could represent an important issue with regard to perinatal care.^16,19^ The study had a power of 87%, with a 0.05 alpha for two-tailed tests.

### Study variables

**Figure 1:**
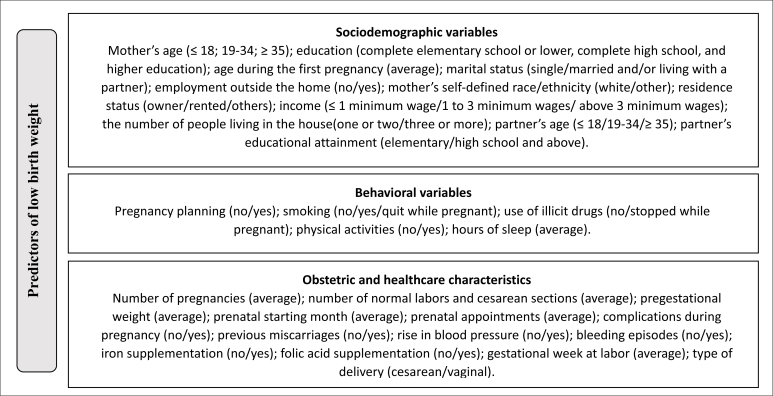
Flowchart of the domains examined as predictors of low birth weight in the current study.

LBW was taken as the dependent variable (DV), according to the World Health Organization criteria, that is less than 2,500 g.^2,3^ DV was obtained from the Live Birth Certificates (in Portuguese, Declaração de Nascido Vivo [DNV]) in the Municipality's Health Secretariat. Independent variables were separated into blocks: sociodemographic, behavioral, and obstetric and healthcare characteristics.^16,20^ Figure 1 presents a flowchart of the domains examined as predictors of low birth weight in the current study.

The first block included the mother's age (≤ 18; 19–34; ≥ 35); educational attainment (complete elementary school or lower, complete high school, and higher education); age during the first pregnancy (average); marital status (single/married and/or living with a partner); employed outside the home (no/yes); mother's self-defined race/ethnicity (white/other); residence status (owner/rented/others); income (≤ 1 minimum wage/1 to 3 minimum wages/above 3 minimum wages); the number of people living in the house (one or two/three or more); partner's age (≤ 18; 19–34; ≥ 35); and partner's educational attainment (elementary/high school and above).^13^

Following a previous study,^20^ the second block of independent variables comprised behavioral data, including pregnancy planning (no/yes); smoking (no/yes/quit during pregnancy); use of illicit drugs (no/stopped while pregnant); physical activity (no/yes); and hours of sleep (average). The third and last block of independent variables included obstetric and healthcare conditions: number of pregnancies (average); number of normal labors and cesarean sections (average); pregestational weight (average); prenatal starting month (average); prenatal appointments (average); complications during pregnancy (no/yes); previous miscarriages (no/yes); rise in blood pressure (no/yes); bleeding episodes (no/yes); iron supplementation (no/yes); folic acid supplementation (no/yes); gestational week at labor (average); and type of delivery (cesarean/vaginal). Pregestational weight in kilograms (kg) and height in meters (m) were collected from women's health documents and used to calculate the pregestational body mass index (kg/m^2^).

### Procedures

This study was approved by the Ethics Committee in Human Research of the Universidade Estadual do Oeste do Paraná on July 02, 2018 (approval no.:2.748.428). Selected by convenience, the sample was composed of pregnant women assisted by the SUS who resided in the city. They were approached while waiting for their prenatal appointments at UBS and invited to answer a questionnaire administered by previously trained researchers (graduate and undergraduate students, all from health-related courses). All women included in the study agreed to participate and signed consent forms. In cases where women were legal minors (less than 18 years old), their legal guardians signed a consent form.

Data on newborns, including sex, weight (g), presence of congenital anomalies, type of delivery, gestational age at birth, number of prenatal appointments, and prenatal starting month, were collected from the DNV. This procedure was authorized by the city's Health Secretariat, specifically its Sanitary Surveillance and Epidemiology sector. The Secretariat also provided data on fetal deaths and abortions.

Infants born alive during twin pregnancies and newborns with congenital anomalies were excluded from the study. When more than two newborns fulfilled the inclusion criteria in the control group, one newborn was randomly selected by drawing lots.

### Data analyses

After completing the questionnaires, the data were tabulated using Microsoft Excel for Microsoft 365 MSO version 2301 (Microsoft Corp., New York, United States). Data were inspected for incorrect or missing information as well as for extreme cases. A 5% limit was adopted for missing data that was not exceeded. For continuous variables, the normality of data was checked using the Shapiro–Wilk test, and significant values were indicative of normality violation. In these cases, comparisons were performed using nonparametric statistics. Welch's t-test was used to compare the means as the low- and normal-weight groups differed in size. For the comparison of categorical variables, the chi-squared tests with and without Yates’ correction for continuity were used. As effect size of bivariate analyses, Cramer's *V* and Cohen's *d* were used. The effect sizes were classified as follows: Cramer's *V* (weak: > 0.05; moderate: > 0.10; strong: > 0.15; very strong: > 0.25) and *d* = 0.20 (small), *d* = 0.50 (medium), and *d* = 0.80 (strong).^21^

Thus, to respond to the first objective, differences in categorical variables among the groups were investigated using the chi-squared tests with and without Yates’ correction for continuity, and Fisher's Exact test, as defined in each case. For the continuous variables presented in Table 1, the Shapiro–Wilk test indicated that only maternal age had a normal distribution. Other variables were compared using Welch's *t* test or nonparametric techniques (Mann-Whitney test).

**Table 1:** Description of sociodemographic, behavioral, obstetric, and healthcare variables of cases and controls (n = 78)

Variables			Cases (n = 26)	Controls (n = 52)	Effect(P value)
			Means ± SDn (%)	Means ± SDn (%)	
**Sociodemographic variables**							
**Woman's age**			26.07 ± 6.24	25.92 ± 5.47	-0.03 (0.91)
	Age (categories)				0.19 (0.17)
		18 years-old or younger	4 (50%)	4 (50%)	
		19–34 years	20 (29.9%)	47 (70.1%)	
		Older than 35	2 (66.7%)	1 (33.3%)	
**Woman's education**					0.09 (0.72)
	Complete elementary school or less		5 (38.5%)	8 (61.5%)	
	Complete or incomplete high school		13 (28.5%)	31 (70.5%)	
	University or more		8 (38.1%)	13 (61.7%)	
**Age in the first pregnancy**			22.38 ± 5.90	21.01 ± 4.82	-0.25 (0.31)
**Marital status**					0.00 (1.00)
	Single		2 (33.3%)	4 (66.7%)	
	Married or living together		24 (33.3%)	48 (66.7%)	
**Works outside home**					
	Yes		13 (29.5%)	31 (70.5%)	0.09 (0.42)
**Woman's race/ethnicity**					0.06 (0.75)
	White		17 (36.2%)	30 (63.8%)	
	Other		9 (30.0%)	21 (70.0%)	
**Living arrangements**					0.07 (0.49)
	Owner		16 (30.8%)	36 (69.2%)	
	Rental/other		10 (38.5%)	16 (61.5%)	
**Income**					0.21 (0.14)
	1 minimum wage or less		5 (35.5%)	8 (61.5%)	
	1 to 3 minimum wages		15 (41.7%)	21 (58.3%)	
	More than 3 minimum wages		6 (20.7%)	23 (79.3%)	
**Area**					0.12 (0.36)
	Urban		23 (35.9%)	41 (64.1%)	
	Rural		3 (21.4%)	11 (78.6%)	
**Number of people in the house**					0.14 (0.19)
	One or two		14 (41.2%)	20 (58.8%)	
	Three or more		12 (27.3%)	32 (72.7%)	
**Partner's age**			30.07 ± 7.32	28.72 ± 6.52	-0.19 (0.41)
**Partner's education**					0.20 (0.13)
	Elementary school or less		9 (50%)	9 (50%)	
	Complete high school or above		16 (27.6%)	42 (72.4%)	
**Behavioral variables**					
**Planned pregnancy**					0.07 (0.69)
	Yes		11 (29.7%)	26 (70.3%)	
**Smoking**					**0.33 (0.01)**
	Yes		3 (100%)	0 (0%)	
	Quit while pregnant		4 (57.1%)	3 (42.9%)	
**Using illicit drugs**					**0.26 (0.04)**
	No		22 (30.1%)	51 (69.9%)	
	Quit while pregnant		4 (80%)	1 (20%)	
**Practice of physical exercise**					0.04 (0.93)
	Yes		9 (31%)	20 (69%)	
**Hours of sleep**			7.96 ± 2.10	7.69 ± 1.90	0.13 (0.58)
**Obstetric and healthcare characteristics**							
**Number of pregnancies**			1.23 ± 1.86	1.51 ± 1.30	0.18 (0.48)
**Normal childbirths**			0.50 ± 1.14	0.41 ± 0.75	-0.09 (0.72)
**Cesarean section**			0.19 ± 0.50	0.32 ± 0.51	0.27 (0.26)
**Pregestational weight**			59.46 ± 12.48	65.00 ± 14.91	0.40 (0.08)
**Pregestational body mass index**			23.07 ± 5.25	24.93 ± 5.93	0.33 (0.16)
**Beginning of prenatal care (month)**			2.56 ± 1.19	2.48 ± 1.23	-0.06 (0.78)
**Prenatal consultations**			8.56 ± 2.26	9.53 ± 2.66	0.37 (0.13)
**Complications in the pregnancy**					0.02 (1.00)
	Yes		7 (33.3%)	14 (66.7%)	
**Previous abortion**					**0.15 (0.25)**
	Yes		1 (12.5%)	7 (87.5%)	
**Increase in blood pressure**					**0.07 (0.76)**
	Yes		4 (26.7%)	11 (73.3%)	
**Bleeding**					**0.04 (0.76)**
	Yes		4 (28.6%)	10 (71.4%)	
**Iron supplementation**					0.11 (1.00)
	Yes		25 (33.3%)	50 (66.7%)	
**Folic acid supplementation**					0.00 (1.00)
Yes			23 (31.9%)	49 (68.1%)	
**Gestational week**			36.84 ± 2.88	38.92 ± 1.54	**0.90 (0.002)**
**Type of labor**					0.15 (0.32)
	Cesarean section		3 (60%)	2 (40%)	
	Vaginal childbirth		23 (31.5%)	50 (68.5%)	

The statistically significant associations are in bold.

To fulfill our second objective, we sought to verify the effects of the independent variables in the LBW outcome through binary logistic regression models, and independent variables with P values of 0.20 or less in bivariate analyses (i.e., Table 1) were inserted. Continuous variables were standardized to improve the interpretation of the results. Variables with fewer than five subjects per cell were excluded from the list of predictors. Results of logistic analyses included the crude odds ratios (OR) and adjusted OR with robust standard errors, standardized coefficients, and 95% bias-corrected and accelerated (BCa) confidence intervals (CI) with bootstrapping (10,000 resamples).^22^ Extreme cases that could compromise the multivariate models were examined using Cook's distance with a tolerance of 1. To select the best explanatory model for logistic regression, the Hosmer-Lemeshow test (cutoff point > 0.05) and the Omnibus Test of Model Coefficients (cutoff point > 0.05) were employed. A smaller Akaike Information Criterion value and increasing explained variance (Nagelkerke's R^2^) were considered when choosing the multivariate final model. Co-variables were established according to a previous study,^6^ which also used DNV and showed that premature births represented a 35 times higher risk of LBW than term births. Thus, gestational age was included in the multivariate data analysis model.

The analyses were carried out in the programs SPSS version 23.0 (IBM Corp., Armonk, New York, United States) and JASP version 0.17.1 (Jasp Team, Amsterdam, The Netherlands), 95% confidence interval (CI) and P values of 0.05 or less were adopted as the criterion of statistical significance. Since all LBW babies born during the study were included and paired by sex and date of birth in a 1:2 ratio, the statistical power was computed *a posteriori*. Thus, G*Power version 3.1.9.7 (Institute for Experimental Psychology, Dusseldorf, Germany) was used, which showed that the study had a power of 87% with 0.05 alpha for two-tailed tests.

## RESULTS

The sample loss included 35 participants; two twins were excluded due to this group's particular characteristics in terms of LBW, five babies were excluded due to congenital anomalies, three due to fetal losses and abortions, and 25 participants because their names were not included in the Health Secretariat's Live Birth Certificates file. Hence, 26 babies were allocated to the experimental group and 52 to the control group.

Regarding sociodemographic variables, Table 1 shows a comparison between the cases and controls. There were no statistically significant differences between the variables in this set. However, statistically significant differences were observed in behavioral and health assistance variables. Thus, the number of smokers or those who quit during pregnancy, as well as users of illegal drugs, was significantly higher among the mothers of babies in the case group—those with LBW. Cramer's *V* pointed that these differences are very strong. Welch's *t* test showed strong, significant differences between gestational weeks, which were smaller among the cases (Table 1).

Subsequently, a logistic regression analysis was performed. Of the five models tested by the forward procedure, the best model is shown in Table 2, having fulfilled all the criteria simultaneously. It maintained two protective factors that explained 36% of the LBW variance with a 0.92 specificity performance diagnosis.

**Table 2:** Logistic regression analyses of factors associated with low weight at birth (n = 78)

Variables		Model 1	Model 2	Model 3
		OR (95% CI)	OR (95% CI)	OR (95% CI)
**Income**				
	1 minimum wage or less	2.40 (0.57, 10.05)	2.46 (0.58, 10.37)	---
	1 to 3 minimum wages	2.74 (0.90, 8.36)	2.84 (0.92, 8.80)	---
	More than 3 minimum wages	1	1	---
**People in the house**				---
	One or two	1	1	---
	Three or more	0.54 (0.21, 1.39)	0.52 (0.20, 1.37)	---
**Father education**				
	Father's education (Elementary)	1	1	1
	Father's education (High school or above)	0.38 (0.12, 1.13)	0.38 (0.12, 1.14)	0.22 (0.06, 0.99)
**Gestational week**		0.24 (0.11, 0.55)	0.24 (0.94, 1.14)	0.17 (0.05, 0.54)
**Pregestational body mass index**		0.70 (0.41, 1.18)	0.68 (0.39, 1.17)	---

Values are expressed as odds ratio (OR) and 95% confidence intervals (95% CI); Model 1 = unadjusted (crude estimates); Model 2 = adjusted for woman's age; Model 3 = adjusted for independent variables with P ≤ 0.05 within the model.

According to the results, the gestational week (OR = 0.12, 95% CI: 0.04–0.52) and fathers’ educational level (high school or above; OR = 0.22, 95% CI: 0.06–0.99) were related to lower chances of low birth weight. Notably, the findings indicate that the strongest predictor was the gestational week, reducing up to 82% the chances of a baby being born with ≤ 2,500 g.

## DISCUSSION

This study aimed to verify the association between LBW and sociodemographic and behavioral factors, as well as obstetric and healthcare characteristics, using a community-based case-control design. Thus, our hypothesis was partially confirmed. We assumed that, in sociodemographic terms, parents’ higher income and education would reduce the chances of LBW,^13^ while risk behavioral factors, such as smoking and drug use, would augment the odds of LBW.^1,10,11^ A second hypothesis was that access to health, demonstrated by an earlier start and a higher number of prenatal care visits, would act as a protective factor for LBW.

Regarding the sociodemographic variables of the pregnant women, we did not find any differences between mothers of babies with LBW and normal-weight newborns. Thus, our income-related hypothesis is yet to be confirmed. Moreover, the average age found in our study was approximately 26 years old, both for the case and control groups—a similar value to those previously reported.^23^ It is known that the “optimal” stage for reproduction is between 19 and 34, and being a mother before or after these periods increases LBW predisposition.^24^ While we did not set any hypotheses about age and LBW's relation, the lack of evidence of such association in our study may be due to a small number of underaged women or those over 35 years.

Pregnancy is a physiological stage during which eating habits are vital for good outcomes. Family income greatly influences pregnancies as it allows access to food and other needs.^13,15^ According to Souza et al.,^25^ more than four times the current minimum wages are needed to cover the average needs of Brazilian families. However, a favorable income does not ensure good food choices or food security. It may even contribute to chronic noncommunicable diseases and complications during pregnancy, such as obesity and diabetes.^4^

In addition, low education is usually reported in the literature as an important variable for LBW, not only when it refers to the mother's education but also partners or other people leading the family.^13^ Notwithstanding, only a few studies associate paternal characteristics with the outcome birth. For instance, recent evidence showed a relationship between low paternal education and prematurity, but did not provide any information regarding possible links with LBW.^26^ Thus, we hypothesized that high education would be a protective factor for LBW.

Confirming our assumption, a partner's education (high school or higher) significantly decreased the likelihood of LBW. Elevated educational attainment might act as a protective factor against LBW during pregnancy, as it increases access to information, and consequently health care, and impacts family income.^6^ The participation of fathers or partners in pregnancy is a subject that involves social and cultural determinants, as the experience of pregnancy is understood differently by the pregnant woman and the partner. Prenatal care contributes to each person's understanding of their roles, responsibilities, and behavioral impacts on new human beings. The conjugal situation, partner's presence, and participation have positive reflexes throughout pregnancy, birth, the baby's stimulus, and acceptance of breastfeeding. Thus, it has direct implications on the pregnant woman's mental health.^27^

Women's behavior and lifestyle may foster physiological disorders during pregnancy, reflecting on the development of the baby after birth.^1,15,28^ Thus, prenatal care is extremely crucial, which might explain why majority of pregnant women in Brazil receive prenatal care, despite barriers.^14,29^ According to Cunha et al.,^14,29^ less than 25% of Brazilian cities meet the criteria of quality prenatal care, and estimations become more critical as the number of inhabitants increases. Inadequate prenatal care is a risk factor for LBW.^23^ Our results showed that mothers in the case group carried out 8.56 prenatal checkups on average, while mothers in the control group had an average of 9.53 checkups. These findings support our hypothesis that a greater adherence to prenatal care decreases the risk of LBW. In addition, the gestational week was significantly associated with LBW, confirming our hypothesis.

Apart from the already discussed hypotheses, this study raised a few additional issues that must be highlighted from a maternal-infant health research perspective. First, LBW rates have been rising worldwide. This phenomenon is derived from changes in women's social roles, which reflect increasing maternal age and search for assisted reproduction techniques.^15^ Thus, LBW is directly related to the access and use of healthcare services. Mesquita-Costa et al.^6^ concluded that fewer than seven prenatal checkups represented a 97% increase in the risk of LBW. However, both cases and controls showed an average number of prenatal checkups close to that suggested in the literature. Therefore, there might be a qualitative rather than a quantitative difference in prenatal care procedures.

Although this study presented data obtained from multiple sources relevant to mothers’ and babies’ health, its limitations must be considered. The case-control design does not allow for the comprehension of clear-cut causal relationships between exposure and dependent variables. Nonetheless, common limitations in this type of study—selection, classification, generalizability, and research biases—were substantially reduced, as the criteria for defining LBW were obtained after the collection of exposure variables.

## CONCLUSION

Our findings confirm previous investigations on LBW's multi-causality, showing that the gestational week could reduce up to 82% chances of a baby being born with ≤ 2,500 grams. This association with paternal education underlines the importance of comprehensive policies protecting newborns, and suggests that the subsequent developmental stages of these babies may be compromised by low paternal education.
